# Immune signatures in variant syndromes of primary biliary cholangitis and autoimmune hepatitis

**DOI:** 10.1097/HC9.0000000000000123

**Published:** 2023-04-26

**Authors:** Christoph Schultheiß, Silja Steinmann, Edith Willscher, Lisa Paschold, Ansgar W. Lohse, Mascha Binder

**Affiliations:** 1 Department of Internal Medicine IV, Oncology/Hematology, Martin-Luther-University Halle-Wittenberg, Halle (Saale), Germany; 2 First Department of Medicine, University Medical Center Hamburg-Eppendorf, Hamburg, Germany; 3 Division of Medical Oncology, University Hospital Basel, Basel, Switzerland

## Abstract

Variant syndromes of autoimmune hepatitis (AIH) and primary biliary cholangitis (PBC) share diagnostic features of both entities, but their immunological underpinnings remain largely unexplored.

**Methods:**

We performed blood profiling of 23 soluble immune markers and immunogenetics in a cohort of 88 patients with autoimmune liver diseases (29 typical AIH, 31 typical PBC and 28 with clinically PBC/AIH variant syndromes). The association with demographical, serological and clinical features was analyzed.

**Results:**

While T and B cell receptor repertoires were highly skewed in variant syndromes compared to healthy controls, these biases were not sufficiently discriminated within the spectrum of autoimmune liver diseases. High circulating checkpoint molecules sCD25, sLAG-3, sCD86 and sTim-3 discriminated AIH from PBC on top of classical parameters such as transaminases and immunoglobulin levels. In addition, a second cluster of correlated soluble immune factors encompassing essentially TNF, IFNγ, IL12p70, sCTLA-4, sPD-1 and sPD-L1 appeared characteristic of AIH. Cases with complete biochemical responses to treatment generally showed a lower level of dysregulation. Unsupervised hierarchical clustering of classical and variant syndromes identified two pathological immunotypes consisting predominantly of either AIH or PBC cases. Variant syndromes did not form a separate group, but clustered together with either classical AIH or PBC. Clinically, patient with AIH-like variant syndromes were less likely to be able discontinue immunosuppressive treatment.

**Conclusions:**

Our analyses suggest that variants of immune mediated liver diseases may represent an immunological spectrum from PBC to AIH-like disease reflected by their pattern of soluble immune checkpoint molecules rather than separate entities.

## INTRODUCTION

Autoimmune hepatitis (AIH) is a severe chronic and relapsing inflammatory liver disease characterized by an ongoing autoimmune reaction directed against hepatic autoantigens.^1^–^3^ Long-term immunosuppression is needed to prevent liver failure^4^–^6^ and progression to advanced liver disease/cirrhosis.^7^–^10^ Primary biliary cholangitis (PBC) is a chronic cholestatic autoimmune liver disease characterized by the destruction of biliary epithelial cells and antimitochondrial antibodies (AMAs) in at least 90% of patients and affects mainly middle-aged and older women.^11^,^12^ Treatment with ursodeoxycholic acid can slow down but not fully stop PBC progression.^12^ PBC/AIH variant syndromes represent variant forms of AIH or PBC that present with nonclassical features such as cholestatic attributes in AIH or elevated transaminases in PBC in combination with autoantibodies and raised IgG levels.^13^–^15^ The manifestation of variant syndromes occurs often sequentially over time with no overlapping features at first diagnosis.^14^ PBC/AIH variant syndromes remain uncommon disorders in ∼5%–20% of patients with diagnostic features of either AIH or PBC.^14^ This wide range reflects the lack of standardization of criteria used to define AIH-PBC overlapping disease. From a biological perspective, it remains unclear whether these syndromes should be considered unique autoimmune entities with damage to bile ducts and hepatocytes or whether they are a presentation of 2 different diseases (PBC and AIH). It has been proposed that variant clinical/laboratory presentations may also result, if PBC is developed in an human leukocyte antigen (HLA) context predisposing to AIH, leading to the term PBC with secondary AIH.^16^

We previously reported a T cell and B cell immune signature of AIH that we found in blood and liver immune cell repertoires by next-generation immunosequencing.^17^ The signature consisted essentially of a strongly biased T cell receptor beta chain (TRB) V gene usage that was independently found in active AIH but also on complete biochemical response and that differed from immune repertoires of patients with PBC. In the work presented here, we focused on a cohort of 28 cases of variant syndromes of PBC or AIH to determine their immune architectures and cytokine deregulation in comparison with 2 cohorts of patients with classical presentation of AIH or PBC. Intriguingly, our study reveals that a set of soluble immune checkpoint molecules more faithfully distinguishes between classical AIH and classical PBC than conventional biomarkers such as transaminases, cholestasis markers, Ig levels, or autoantibodies. Unsupervised hierarchical clustering shows that variant syndromes do not show a distinctive immune fingerprint but cluster either together with classical AIH or classical PBC. These data may lay the foundation for a new understanding of the immunological continuum between AIH and PBC and suggest a clinical role for immunoprofiling at this crossroad of diseases.

## METHODS

### Patient characteristics

Peripheral blood of patients with classical AIH (n=29), classical PBC (n=31), and variant syndromes of AIH or PBC (n=28) was collected in EDTA monovette tubes (Sarstedt) between 2017 and 2019 during routine clinical monitoring at the University Medical Center Hamburg-Eppendorf. Patients with AIH were diagnosed according to the simplified diagnostic criteria of the International Autoimmune Hepatitis Group,^18^ combined with a response to immunosuppressive therapy. PBC was diagnosed according to the European Association for the Study of the Liver (EASL) guidelines: persistent elevation of cholestasis parameters and either presence of PBC-specific autoantibodies (AMA-M2, or sp-100/gp210 AMAs) or PBC-typical histology. Variant syndromes of PBC and AIH were defined as patients fulfilling diagnosis criteria of PBC but further showing features of AIH, objectified by substantial histological periportal-based inflammation (modified Hepatitis Activity Index≥5).^19^ Demographic, clinical, and serological characteristics of the patients at the time point of inclusion in our study are summarized in Table 1. Most patients with AIH received azathioprine/thiopurines or a combination with low-dose prednisolone as first-line therapy. Eight patients received low-dose corticosteroids as monotherapy owing to intolerance to thiopurines. Nearly all patients with PBC were treated with ursodeoxycholic acid (UDCA) as first-line therapy. One patient had discontinued UDCA owing to intolerance at the time of study inclusion, and one patient additionally received bezafibrate. Three patients with variant syndrome initially received second-line treatment for PBC. Clinical follow-up data of these patients were collected from 2017 to 2022. The clinical course of the patients was monitored with yearly outpatient clinic visits, including clinical, laboratory, and elastographic workups. Patients with follow-up time of <2 years were excluded. Subsequent to reaching a clinical end point (liver transplantation or liver-related death), patients were excluded from the follow-up.

**TABLE 1:** Demographical, serological, and clinical characteristics of patients with AIH, PBC, and VS

	AIH (n=29)	PBC (n=31)	VS (n=28)	Total (N=88)	*p*
Age [median (range)] (y)	58 (20–79)	57 (27–77)	54 (28–77)	NS	0.699
Sex				NS	0.641
Female	25 (86.2)	29 (93.5)	25 (89.3)		
Male	4 (13.8)	2 (6.5)	3 (10.7)		
AST	29 (12–97)	29 (15–119)	32 (13–88)	NS	0.73
ALT	31 (15–90)	35 (18–77)	35 (11–84)	NS	0.621
GGT	44 (13–153)	76 (14–460)	91 (14–410)	NS	0.061
ALP	87 (36–184)	135 (44–436)	127 (40–308)	0.006	0.006
Bilirubin	0.8 (0.1–2.4)	0.4 (0.2–1.0)	0.5 (0.3–1.1)	0.002	0.002
IgG	13.3 (7.2–49.6)	11.4 (7.0–19.2)	14.3 (6.1–22.7)	NS	0.108
IgA	2.2 (0.5–4.9)	2.1 (0.6–5.0)	2.4 (0.5–6.1)	NS	0.728
IgM	1.5 (0.3–10.7)	2.2 (0.2–8.7)	2.2 (0.4–8.2)	NS	0.215
Anti-ANA	22 (75.9)	74.2 (23)	19 (67.6)	NS	0.774
Anti-AMA	1 (3.4)	74.2 (23)	18 (64.3)	0.0001	0.0001
Anti-SMA	12 (41.1)	6.5 (2)	12 (42.9)	0.002	0.002
Anti-SLA	2 (6.9)	0	1 (3.6)	NS	0.338
Anti-LKM	2 (6.9)	0	2 (2.3)	NS	0.125
Mean transient elastography (kPa)	8.9 (2.6–34.8)	5.1 (2.6–10.3)	8.0 (3.1–22.8)	0.013	0.013
Cirrhosis	27.5 (8)	0	14.3 (4)	0.006	0.006
Fibrosis treatment	27.5 (8)	29.0 (9)	50.0 (14)	NS	0.124
Immunosuppression	93.1 (27)	3.2 (1)	60.7 (17)	0.0001	0.0001
Thiopurines	65.6 (19)	0	53.6 (15)		
Corticosteroids	48.3 (14)	0	32.1 (9)		
Combination of thiopurines and steroids	34.5 (10)	0	21.4 (6)		
UDCA	3.4 (1)	87.1 (27)	92.9 (26)	0.0001	0.0001
Second-line/third-line therapy	0	3.2 (1)	10.7 (3)		

Categorical variables are expressed as n (%). Quantitative variables are described as means with their SDs. The Pearson χ^2^ test and Fisher exact test were used to test difference between categorical variables between more groups. Differences of continuous variables between 2 or more groups were tested using Wilcoxon-Mann-Whitney test or Kruskal-Wallis test, as appropriate.

Abbreviations: ALP, alkaline phosphatase; ALT, alanine aminotransferase; AMA, antimitochondrial antibody; ANA, antinuclear antibody; AST, aspartate aminotransferase; GGT, gamma-glutamyl transferase; LKM, liver-kidney microsomal antibody; NS, not significant; SLA, soluble liver antigen; SMA, smooth muscle antibody; UDCA, ursodeoxycholic acid; VS, variant syndrome.

Complete biochemical response (CBR) of AIH was defined by normalization of serum transaminases (aspartate aminotransferase and alanine aminotransferase) and IgG at any point during treatment in analogy to international consensus.^20^ On clinical and laboratory follow-up, therapy response was controlled by measurement of transaminases, Igs, and cholestasis parameters. A loss of adequate therapy response was defined when patients with AIH and variant syndrome failed to meet criteria of complete biochemical remission. Therapy response in patients with PBC was defined as inadequate when patients did not meet Paris II-criteria (alkaline phosphatase 1.5<upper limit of normal or aspartate aminotransferase 1.5<upper limit of normal or bilirubin<1 mg/dL).^21^

Progression of fibrosis was checked yearly by noninvasive liver stiffness measurement by transient elastography (FibroScan; Echosense). Values >6 kPa marked presence of fibrosis, values >16 kPa indicated liver cirrhosis,^22^–^24^ and value increases >5 kPa were considered as progression of fibrosis.^25^ In addition, blood samples were collected from healthy control individuals [healthy donor (HD), n=30]. Written informed consent was obtained from each patient and healthy individual included for the use of their biological material as approved by the ethics commission Hamburg (Ethikkommission der Ärztekammer; project number PV4081). The study was performed in accordance with the declaration of Helsinki of 1975.

### Isolation of peripheral blood mononuclear cells and genomic DNA

Peripheral blood mononuclear cells were isolated by density-gradient centrifugation using Biocoll separation solution (Biochrom AG). Genomic DNA from peripheral blood mononuclear cells was isolated with the GenElute mammalian genomic DNA miniprep kit (Sigma-Aldrich) according to the manufacturer’s instructions.

### Next-generation sequencing (NGS) immunosequencing and data analysis

Bulk TRB and IGH immunosequencing was performed as described.^17^,^26^,^27^ In short, rearranged TRB and IGH loci were amplified from 250 to 500 ng genomic DNA using the BIOMED2-TRB-B and BIOMED2-FR1 primer pools and the Phusion HS II polymerase system (Thermo Fisher Scientific Inc.). Amplified fragments were tagged with Illumina-compatible adapters and barcoded for sample identification. Final amplicons were purified using the NucleoSpin Gel and PCR Clean-up kit (Macherey-Nagel), quantified on the Qubit platform (Qiagen), and pooled to a final concentration of 8 nM. Pool quality was assessed using an Agilent 2100 Bioanalyzer (Agilent Technologies). Sequencing and demultiplexing was performed on an Illumina MiSeq sequencer (600-cycle single indexed, paired-end run, V3-chemistry). TRB and IGH rearrangements were aligned to reference genomes (default library for TRB, IMGT library v3 for IGH) using MiXCR.^28^ Each unique complementarity-determining region (CDR3) nucleotide sequence was defined as one clone, whereas nonproductive reads and sequences with <2 read counts were discarded. Analysis of global immune repertoire metrics (diversity, richness, somatic hypermutation) and V-J gene usage as well as data plotting was performed in RStudio (version 1.3.959) as described in other studies.^17^,^26^,^27^,^29^

### Cytokine profiling

Plasma was isolated by centrifugation of the whole blood for 15 minutes at 2000*g*. Samples were stored at −80°C until use. Cytokine plasma levels were quantified using the LEGENDplex Human B Cell Panel (13-plex) and the Human Immune Checkpoint Panel 1 (10-plex) from BioLegend according to the manufacturer’s suggestions. Data were acquired using the BD FACSCelesta flow cytometer and analyzed with BioLegend LEGENDplex software. Correlations were calculated using the R package corrplot.

### Statistical analysis

Differences in NGS metrics and cytokine levels were studied by the Student *t* test. All statistical analyses have been performed with GraphPad Prism 8.0.2 (GraphPad Software). Demographical, clinical, and laboratory features were analyzed with IBM SPSS Statistics. The Pearson χ^2^ test and Fisher exact test were used to test difference of categorical variables between 2 or more groups. Differences of continuous variables between two or more groups were tested using Wilcoxon-Mann-Whitney-Test or Kruskal-Wallis-Test, as appropriate, and SPSS Statistics (IBM SPSS Statistics for Windows, Version 27.0, released 2020. Armonk, NY: IBM Corp). Correlations were calculated using the R package corrplot and RStudio version 3.5.1. Graph Pad Prism version 8.3.1 and Adobe Illustrator version 24.1.1 and were used for all other statistical analyses and data plotting.

## RESULTS

### Deep repertoire sequencing of patients with variant syndromes of AIH or PBC

**FIGURE 1:**
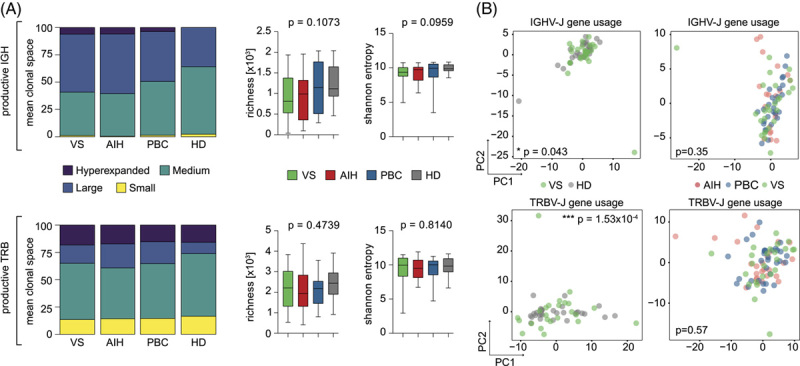
Immune repertoire metrics and V-J gene usage. (A) Broad metrics of the productive IGH and TRB repertoires were compared between VS (n=28), AIH (n=29), and PBC (n=31) patient samples, and HDs (n=30). Statistical analysis: ordinary 1-way ANOVA for blood samples, and unpaired 2-tailed Student *t* test for liver samples. (B) PCA of productive TRB and IGH V-J gene usage in the peripheral blood of patients with VS, AIH, and PBC. Abbreviations: AIH, autoimmune hepatitis; HD, healthy donor; IGH, immunoglobulin heavy locus; PBC, primary biliary cholangitis; PCA, principal component analysis; TRB, T cell receptor beta locus; VS, variant syndrome.

To gain deeper insights into the adaptive immune architecture of variant syndromes of AIH and PBC, we performed immuno-NGS to analyze peripheral blood T cell and B cell repertoires in our cohort of 28 variant syndrome cases. Control data sets were available from AIH, PBC, and HD cohorts.^17^,^27^ Clinical and laboratory characteristics of the disease cohorts are shown in Table 1. Patients with variant syndrome shared serological features of both AIH and PBC. Most patients with AIH were treated with azathioprine/thiopurines or a combination with low-dose prednisolone as first-line therapy and almost all patients with PBC with UDCA. The majority of the patients with variant syndrome received both UDCA and immunosuppressive treatment. Hepatic inflammation displayed by elevation of serum liver enzymes did not significantly differ between the groups. We observed no differences in global immune metrics specific to patients with variant syndromes (Figure 1A). In our previous study, we showed biased TRB and IGH V-J gene usage in blood-derived and liver-derived T and B cells in classical AIH or PBC versus HD (with only minor differences between AIH and PBC).^17^ Likewise, principal component analysis showed skewing of TRB and IGH V-J gene usage in patients with variant syndromes relative to HD (Figure 1B) comparable to that previously observed for classical AIH and PBC.^17^ Multivariate ANOVA (Pillai-Bartlett trace test) suggested that variant syndromes had repertoire biases that cannot be distinguished from those of classical AIH and PBC (Figure 1B).

### Differential profiles of cytokines and soluble immune checkpoints in classical AIH, PBC, and variant syndromes

**FIGURE 2:**
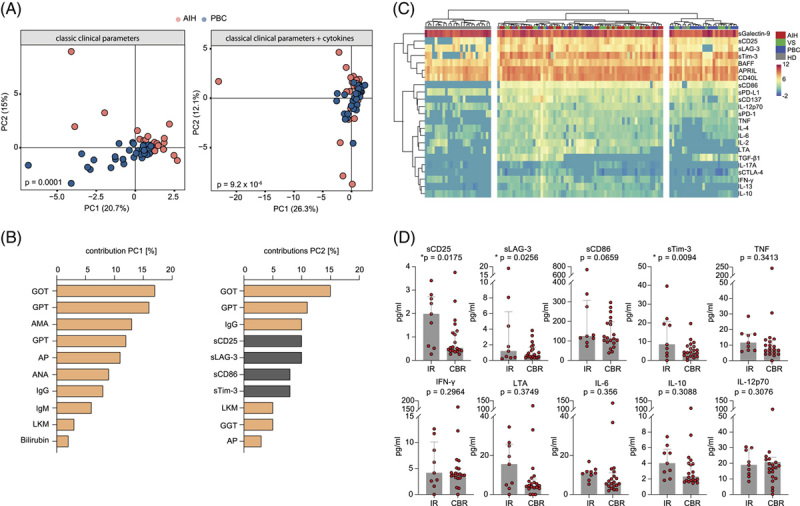
Discriminative serological markers and plasma cytokine levels in AIH and PBC. (A) PCA of classical serological markers, clinical parameters, cytokines, and soluble immune checkpoints in patients with AIH (n=29) and PBC (n=31). (B) Contributions to the indicated principal components from (A) are shown. Classical clinical parameters are shown in yellow ocher and cytokines or soluble checkpoints in gray for visualization purposes. (C) Unsupervised hierarchical clustering (Ward D2, Canberra distance) of log10 transformed cytokine concentrations in plasma samples of patients with VS (n=28), patients with AIH (n=29), and patients with PBC (n=31) or healthy individuals (HDs; n=30). (D) Median plasma concentration of selected cytokines in patients with AIH with CBR (n=20) and IR (n=9). Error bars indicate interquartile range. Statistics: Student *t* test. Abbreviations: AIH, autoimmune hepatitis; ALP, alkaline phosphatase; ALT, alanine aminotransferase; AMA, antimitochondrial antibody; ANA, antinuclear antibody; AST, aspartate aminotransferase; CBR, complete biochemical response; GGT, gamma-glutamyl transferase; GOT, glutamic oxaloacetic transaminase; GPT, glutamate-pyruvate transaminase; HD, healthy donor; IR, insufficient response; LKM, liver-kidney microsomal antibody; LTA, lymphotoxin alpha; PBC, primary biliary cholangitis; PCA, principal component analysis; VS, variant syndrome.

As immunogenetic features were good disease separators but less robust for individual disease subsets, we set out to identify other parameters with higher discriminative power. For this purpose, we profiled the plasma levels of 13 typical cytokines involved in immune cell regulation, class switch recombination, IgG secretion, or B cell and T cell interplay as well as a set of 10 soluble (s) immune checkpoints. We first tested how robust these factors differentiate between AIH and PBC as compared with established clinical biomarkers such as liver enzymes, cholestasis parameters, autoantibodies, and Ig levels. To do this, we performed a principal component analysis using all classical clinical parameters and laboratory markers. As to be expected, this analysis showed significant differences between the subgroups, with transaminases, AMA, ANA, IgG, and IgM being the strongest separators (Figure 2A and B, *p*=0.0001). We then repeated the principal component analysis including the cytokine and soluble immune checkpoint data. This analysis showed a significantly improved separation of both groups (Figure 2A and B, *p* = 9.2×10^−6^). The most important discriminators were sCD25, sLAG-3, sCD86, and sTim-3 in addition to transaminases and IgG (Figure 2A and B).

Thereafter, we performed unsupervised clustering of the cytokine and soluble immune checkpoint data in HD, AIH, PBC, and variant syndromes to characterize their profile in these subsets. This analysis resulted in 3 distinct clusters, one comprising most of the healthy samples, another most AIH samples, and a third cluster was enriched in PBC samples (Figure 2C). All 28 samples from patients with variant syndromes clustered either together with the AIH (20/28) or the PBC (8/28) samples, suggesting that variant syndromes may cover an immunological spectrum from AIH-like to PBC-like cases. Within this spectrum, patients with AIH had the highest mean of sCD25, sLAG-3, sCD86, sTim-3, TNF, IFN-γ, IL-6, IL-10, IL-12p70, IL-17A, sCD137 (s4-1BB), and sCTLA-4 levels, whereas PBC samples were characterized by the highest mean IL-2, TGF-β1, and LTA (TNF-β) levels. The mean levels of IL-13, BAFF, APRIL, sCD40L, sPD-L1, and sPD-1 were evenly elevated across disease groups, whereas the mean plasma level of sGalectin-9 showed no elevation in either group as compared with the healthy controls. Interestingly, the disease-specific clusters appeared to be quite robust and to some extent unaffected by disease activity as our samples were derived from patients with different levels of disease control. To explore this in more detail, we separated all classical AIH cases into subcategories of patients meeting the criteria of CBR (defined by normalized transaminases and IgG levels) and with insufficient response to treatment, which failed to reach CBR. Figure 2D highlights the most disease-discriminative markers in addition to exemplary dysregulated cytokines as a function of disease activity. These data show that sCD25, sLAG-3, sCD86, and sTim-3 correlated most with the level of disease control. Notably, especially levels of sCD25 and sLAG-3 remained clearly discriminative when comparing patients with AIH and PBC with CBR (*p* = 0.04 and 0.05, respectively).

### Correlation analysis of clinical parameters, cytokines, and soluble immune checkpoints in classical AIH, PBC, and variant syndromes

**FIGURE 3:**
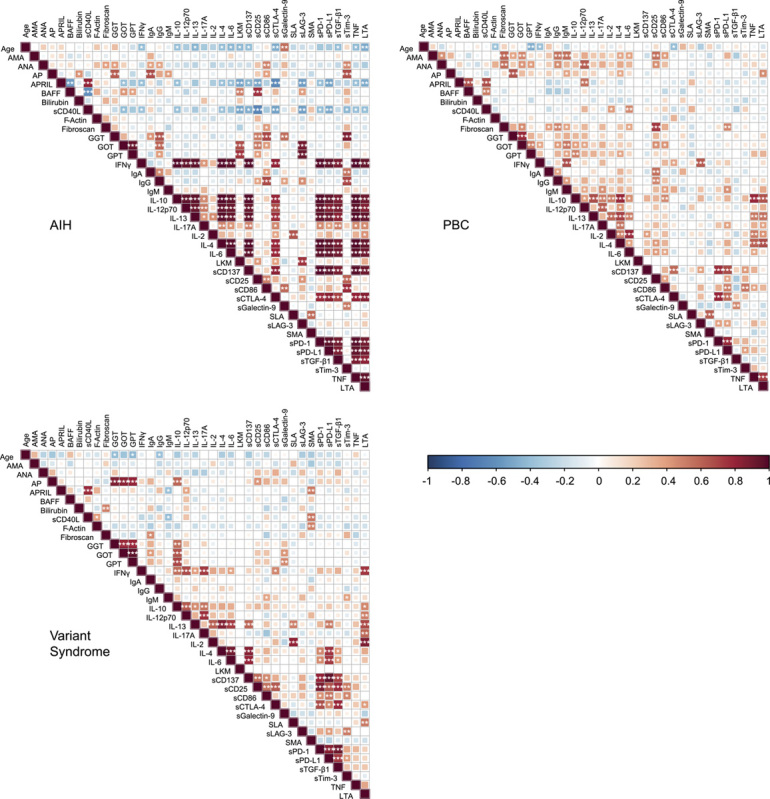
Correlation of clinical parameters and cytokine plasma levels in VS, AIH, PBC, and HD samples. Correlation matrix of all plasma cytokines for the VS (n=28), AIH (n=29), PBC (n=31), and healthy individual (HD; n=30) groups. The ranges of the *p* values for the correlations are indicated with asterisks: *p < 0.05; **p < 0.01; ***p < 0.001. Abbreviations: AIH, autoimmune hepatitis; ALP, alkaline phosphatase; ALT, alanine aminotransferase; AMA, antimitochondrial antibody; ANA, antinuclear antibody; APRIL, proliferation-inducing ligand; AST, aspartate aminotransferase; BAFF, B cell activating factor; CD, cluster of differentiation; CTLA-4, cytotoxic T-lymphocyte–associated protein 4; GGT, gamma-glutamyl transferase; GOT, glutamic oxaloacetic transaminase; GPT, glutamate-pyruvate transaminase; HD, healthy donor; IFN, interferon; LAG-3, lymphocyte-activation gene 3; LKM, liver-kidney microsomal antibody; LTA, lymphotoxin alpha; PBC, primary biliary cholangitis; PD-1, programmed cell death protein 1; PD-L1, programmed death-ligand 1; s, soluble; SLA, soluble liver antigen antibody; SMA, smooth muscle actin; Tim-3, T cell immunoglobulin and mucin domain 3; VS, variant syndrome.

**FIGURE 4:**
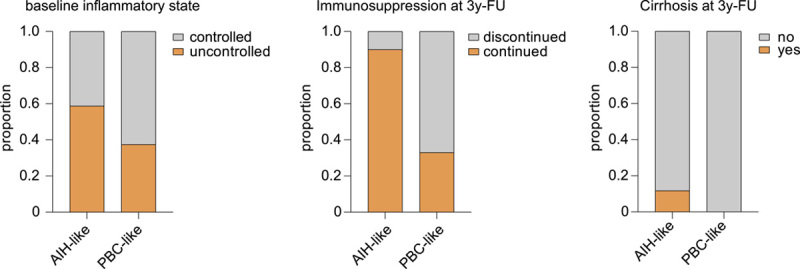
Patient outcome with respect to cytokine profiles. FU data (3 y) for patients with VS with AIH-like cytokine profile (n=17) and PBC-like cytokine profile (n=8). Abbreviations: AIH, autoimmune hepatitis; FU, follow-up; PBC, primary biliary cholangitis; VS, variant syndrome.

Given the robustness of the AIH-like and PBC-like clusters, we next asked whether there are correlating cytokine sets within each disease group that might hint toward a potential disease mechanism. We also included the clinical parameters to explore their relation to cytokine levels. This analysis revealed clusters of soluble factors showed strong correlation with each other in AIH, whereas correlations were more restricted in variant syndromes and PBC (Figure 3). In AIH, these positive correlations could be roughly grouped into 2 blocks. One block encompassed TNF, IL-4, IL-6, IL-10, IL-12p70, IL-13, IL-17A, IFN-γ, LTA, TGF-β1, sCD137, sCTLA-4, sPD-L1, and sPD-1. The other block encompassed sCD25, sLAG-3, sCD86, sTim-3m, and BAFF (Figure 3). Interestingly, a unique negative correlation pattern was found for APRIL and BAFF in AIH (Figure 3). A clear positive correlation of TNF with any other cytokine was not detected in variant syndromes but in PBC, where it correlated with IL-2, IL-4, IL-6, IL-10, IL-13, and LTA (Figure 3). Notably, sCD25 levels also correlated with sCD137, sPD-L1, sCTLA-4, and TGF-β1 in variant syndromes (Figure 3), (Figure 4).

### Clinical significance of the AIH-like and PBC-like immune clusters

To investigate if the AIH-predominant and PBC-predominant immunotypes characterized patients with distinct clinical presentations and outcomes, we analyzed our categorized cases with respect to rates of loss of therapy response, need for second-line and third-line therapy, their potential to discontinue immunosuppressive treatment, and their rate of liver cirrhosis and fibrosis at 3 years of follow-up (Tables 2–4). Patients were followed by yearly clinical, laboratory, and elastographic workups. Patients with follow-up period of <2 years were excluded. Two patients with AIH were excluded after reaching clinical end points (liver-related death and liver transplantation owing to acute liver failure on AIH flare). Follow-up data were assessable for >90% of the initial study population (Figure S1, http://links.lww.com/HC9/A242). The mean follow-up time was 38 months and did not significantly differ between the groups. There were no differences in baseline characteristics of the patients with AIH, PBC, and variant syndrome with available follow-up data (Table 2) compared with the characteristics of the initial patient cohorts as described (Table 1).

**TABLE 2:** Demographical, serological, and clinical characteristics of patients with VS according to their immunogenic phenotype

	AIH-like VS (n=19)	PBC-like VS (n=8)	Total (N=27)	*p*
Age [median (range)] (y)	58.9 (28–77)	57.5 (36–71)	NS	0.389
Sex			NS	0.233
Female	84.2 (16)	100 (8)		
Male	3 (15.8)	0		
AST	30 (13–82)	42 (17–88)	NS	0.238
ALT	34 (13–55)	41 (11–84)	NS	0.735
GGT	101 (14–410)	68 (17–172)	NS	0.515
ALP	126 (40–308)	131 (70–297)	NS	0.979
Bilirubin	0.6 (0.3–1.1)	0.5 (0.3–1.0)	NS	0.449
IgG	15.0 (8.0–22.7)	12.3 (6.1–19.2)	NS	0.106
IgA	2.6 (1.1–6.1)	1.8 (0.5–1.2)	NS	0.106
IgM	2.2 (0.8–3.3)	2.3 (0.4–8.2)	NS	0.217
Anti-ANA	12 (63.2)	6 (75.0)	NS	0.551
Anti-AMA	12 (63.2)	6 (75.0)	NS	0.551
Anti-SMA	8 (42.1)	3 (37.5)	NS	0.824
Anti-SLA	1 (5.3)	0	NS	0.5
Anti-LKM	0	0		
Mean elastography (kPa)	9.0 (4.2–22.8)	6.5 (3.1–12.1)	NS	0.187
Cirrhosis	3 (15.8)	1 (12.5)	NS	0.826
Fibrosis	11 (57.9)	3 (37.5)	NS	0.324
Treatment				
Immunosuppression	10 (52.6)	6 (75.0)	NS	0.28
UDCA	18 (94.7)	8 (100)	NS	0.51
CBR	7 (36.8)	5 (62.5)	NS	0.212

Categorical variables are expressed as n (%). Quantitative variables are described as means with their SDs. The Pearson χ^2^ test and Fisher exact test were used to test difference between categorical variables between more groups. Differences of continuous variables between 2 or more groups were tested using Wilcoxon-Mann-Whitney test or Kruskal-Wallis test, as appropriate.

Abbreviations: ALP, alkaline phosphatase; ALT, alanine aminotransferase; AMA, antimitochondrial antibody; ANA, antinuclear antibody; AST, aspartate aminotransferase; CBR, complete biochemical remission; GGT, gamma-glutamyl transferase; LKM, liver-kidney microsomal antibody; NS, not significant; SLA, soluble liver antigen; SMA, smooth muscle antibody; UDCA, ursodeoxycholic acid; VS, variant syndrome.

Loss of adequate therapy response on follow-up was significantly higher in patients with AIH and variant syndrome compared with patients with PBC. Introduction of second-line or third-line treatment was significantly more frequent in patients with variant syndrome compared with patients with AIH or PBC. The liver stiffness in patients with AIH and variant syndrome was higher compared with patients with PBC at the time of study enrollment. However, on follow-up, median liver stiffness decreased in the AIH patient group, whereas patients with variant syndrome had significantly increased liver stiffness and increased prevalence of liver cirrhosis compared with patients with AIH and PBC (Tables 3 and 4, Figure 4).

**TABLE 3:** Clinical characteristics of patients with AIH, PBC, and VS at study inclusion and upon follow-up

	AIH (n=26)	PBC (n=28)	VS (n=26)	Total (N=80)	*p*
Time to follow-up [median (range)]	36 (12–48)	38 (24–48)	39 (24–48)	NS	0.339
Mean transient elastography (kPa)	8.4 (2.6–34.8)	5.1 (2.6–10.3)	8.3 (3.1–22.80)	0.016	0.016
Cirrhosis	4 (16.7)	0	4 (16.7)	0.074	0.074
Fibrosis	7 (26.9)	8 (28.6)	14 (53.8)	NS	0.119
Upon follow-up					
Loss of therapy response	11 (42.3)	14.9 (4)	65.4 (17)	0.0005	0.0005
Second-line or third-line treatment	0	4 (14.3)	9 (34.6)	0.002	0.002
Withdrawal of immunosuppressive therapy	4 (15.4)	NA	6 (23.1)	NS	0.354
Mean transient elastography (kPa)	7.3 (3.6–20.9)	5.5 (3.1–5.5)	9.4 (3.6–27.00)	0.025	0.021
Cirrhosis	3 (11.6)	0	7 (26.9)	0.015	0.015
Fibrosis	10 (28.4)	10 (35.7)	9 (34.6)	NS	0.67
Progression of fibrosis (>5 kPa)	1 (3.8)	0	4 (15.4)	0.049	0.049
Improvement of fibrosis (>5 kPa)	3 (11.5)	0	1 (3.8)	NS	0.147

Categorical variables are expressed as n (%). Quantitative variables are described as means with their SDs. The Pearson χ^2^ test and Fisher exact test were used to test difference between categorical variables between more groups. Differences of continuous variables between 2 or more groups were tested using Wilcoxon-Mann-Whitney test or Kruskal-Wallis test, as appropriate.

Abbreviations: AIH, autoimmune hepatitis; NA, not available; NS, not significant; PBC, primary biliary cholangitis; VS, variant syndrome.

**TABLE 4:** Clinical characteristics of patients with VS according to their immunogenic phenotype at study inclusion and upon follow-up

	AIH-like VS (n=18)	PBC-like VS (n=7)	Total (N=25)	*p*
Time of follow-up [median (range)]	40 (19–72)	37 (20–46)	NS	0.886
At study inclusion				
Mean transient elastography (kPa)	8.7 (2.6–34.8)	6.5 (3.1–12.1)	NS	0.285
Cirrhosis	3 (17.6)	1 (12.5)	NS	0.957
Fibrosis	9 (50)	2 (33.3)	NS	0.269
Upon follow-up				
Mean transient elastography (kPa)	9.1 (3.6)	10.3 (4.4–20.9)		
Cirrhosis	5 (27.8)	2 (28.6)	NS	0.91
Fibrosis	6 (33.3)	3 (42.8)	NS	0.436
Progression of fibrosis (>5 kPa)	2 (11.1)	2 (28.6)	NS	0.315
Improvement of fibrosis (>5 kPa)	0	1 (12.5)	NS	0.111
Loss of therapy response	14 (82.3)	3 (42.9)	NS	0.143
Second-line or third-line treatment	3 (33.3)	2 (28.6)	NS	0.751
Withdrawal of immunosuppressive therapy	2/13 (15.4)	4/6 (66.6)	0.046	0.046

Categorical variables are expressed as n (%). Quantitative variables are described as means with their SDs. The Pearson χ^2^ test and Fisher exact test were used to test difference between categorical variables between more groups. Differences of continuous variables between 2 or more groups were tested using the Wilcoxon-Mann-Whitney test or Kruskal-Wallis test, as appropriate.

Abbreviations: AIH, autoimmune hepatitis; NS, not significant; PBC, primary biliary cholangitis; VS, variant syndrome.

Thereafter, we searched for clinical parameters that correlated with the AIH-like and PBC-like immunotypes of patients with variant syndrome. Overall, 82% of patients with AIH-like variant syndrome showed a loss of adequate therapy response during follow-up (Table 4, Figure 4). Loss of therapy response appeared less frequent in patients with PBC-like variant syndrome. A milder clinical course of PBC-like variant syndrome was also reflected by a significantly reduced need for immunosuppressive therapy (Figure 4). Immunosuppressive treatment could be discontinued only in 2 individuals of the AIH-like variant syndrome group, whereas the majority of the patients with PBC-like variant syndrome could stop immunosuppressive drugs (Table 4).

## DISCUSSION

Our current understanding of the principal pathobiological mechanism underlying AIH and PBC is that of a T cell–mediated autoimmune response directed against liver autoantigens with a differential pattern of destruction.^1^,^3^ Variant syndromes share clinicopathological aspects of both entities, but the immunological background of such syndromes has been largely unexplored.^14^

To gain deeper insights into the immune dysregulation in variant syndromes of PBC and AIH, we performed NGS-based sequencing to analyze T cell and B cell receptor repertoires in 28 patients with such syndromes and compared these with the repertoires of patients with classical AIH or PBC. We found clear evidence that variant syndromes showed immunogenetic overlaps with both AIH and PBC. More noteworthy, however, were our analyses of 23 cytokines and soluble immune checkpoint molecules. We found that certain markers, especially sCD25, sLAG-3, sCD86, and sTim-3, may represent important discriminators between AIH and PBC that could be used to complement established clinical and laboratory parameters such as liver function tests, autoantibodies and Ig levels. We also found that based on these immunological markers patients, with variant syndromes either cluster together with AIH or PBC, but not with healthy controls, confirming that AIH and PBC have a different immunological underpinning and that variant syndromes may either have an AIH-like or a PBC-like immunotype.

We observed that the majority of patients with variant syndrome had AIH-like signatures. The AIH-like immunotype was characterized by a more prominent inflammatory cytokine signature mirrored by the highest levels of TNF, IL-6, IL-12p70, IL-17A, and IFN-γ in line with AIH data from others.^30^–^32^ In addition, patients with AIH exhibited the highest levels of sCTLA-4, which can contribute to break tolerance and promote inflammation by interfering with inhibitory T cell signaling^33^ and high levels of sCD86, which can activate CD8^+^ T cells and potentiate recall responses by increasing the frequency of INF-γ-producing memory T cells after antigenic trigger.^34^ Considering that variant syndromes are primarily characterized as PBC cases with secondary AIH features,^13^,^14^ the AIH-like cytokine profile of patients with variant syndrome may be interpreted in light of their increased inflammatory activity. Therefore, the corresponding cytokines can be viewed as major drivers of AIH pathogenesis with TNF emerging as a key player. This hypothesis is supported by the efficacy of anti-TNF treatment in patients with refractory difficult-to-treat AIH 35 and the intrahepatic enrichment of autoreactive CD4^+^ T cells with T_H_1 polarization that drive AIH by secreting TNF and IFN-γ.^36^–^39^ Notably, the normalization of liver transaminases and Ig levels in our cohort was accompanied by a decrease of TNF but not IFN-γ and IL-12p70 plasma levels. IL-12p70 is mainly secreted by professional antigen-presenting cells and mediates differentiation and maintenance of the T_H_1 state.^40^ Therefore, these data may indicate that although TNF antagonism can alleviate AIH symptoms, autoreactive CD4^+^ T cells with T_H_1 state may persist with the inflammatory microenvironment sustaining their polarization. This hypothesis is also in line with the high rates of relapsing AIH cases.^1^

Interestingly, we also observed high levels of IL-10 in active AIH that showed a trend toward normalization in controlled disease. IL-10 mainly derives from regulatory and T_H_2 T cells and is considered a prototypical anti-inflammatory cytokine that mediates the reestablishment and maintenance of immune homeostasis.^41^,^42^ In this notion, the high levels in acute AIH might explain the observation of spontaneous counterregulation of T cell autoreactivity,^43^ as well as the normal IgG levels in some patients with very early phases of acute AIH.^6^ In addition, chronic IL-10 elevation might contribute to hypergammaglobulinemia and autoantibody production.^41^,^42^,^44^ Although the described proinflammatory cytokine signatures mirror the inflammatory spectrum of AIH, variant syndromes, and PBC, our multivariate analysis of clinical parameters that are commonly used for diagnosis of autoimmune liver disease combined with cytokine plasma levels revealed that levels of sCD25, sLAG-3, sCD86, and sTim-3 are a distinctive feature of AIH. Notably, the plasma levels of these factors strongly correlated in AIH and to some degree also in variant syndromes but not in PBC, thus suggesting important roles for the development of AIH. Interestingly, the AIH-specific correlation of elevated sCD25, sLAG-3, sCD86, and sTim-3 further corroborates the importance of T_H_1 cells in AIH and also points toward a key role of regulatory T cells (Tregs). In this context, it is interesting to note that the observed elevated sCD25 levels may also serve as a sink for IL-2. As Tregs are highly dependent on exogenous IL-2,^45^ excessive sCD25 may reduce suppressive Treg function in AIH.^46^

Tim-3 was first described as a T_H_1-specific inhibitory surface molecule,^47^ but its expression on other T cell subsets, especially tissue-resident Tregs, as well as on innate immune cells has been described since then.^48^,^49^ Tim-3 mediates Treg supressor functions in an AIH model^50^ and can induce tolerance and the contraction of T_H_1 effector populations.^51^ It is described to be increased in the sera of patients with AIH compared with patients with PBC and associated with disease severity.^52^ The function of soluble Tim-3 is less established. Although it was first discovered in mice as secreted splice variant,^53^ it can also be shedded from the cell surface.^54^ In mouse models, administration of sTim-3 induced hyperproliferation and spontaneous cytokine secretion of T_H_1 cells even in the absence of antigen^53^ or accelerated disease in a model for diabetes by dampening of the antigen-specific immunosuppressive Treg function on autoreactive T_H_1 cells.^55^ Considering these observations, it is plausible that sTim-3 also acts through these mechanisms to regulate T_H_1/Treg function and promote inflammation in AIH as it has also been proposed for systemic lupus erythematosus^56^ and rheumatoid arthritis.^57^

Similar to Tim-3, LAG-3 has been mainly described as co-inhibitory receptor limiting T cell responses, although its ligand-dependent regulatory mechanisms remain somewhat enigmatic.^58^,^59^ Constitutive LAG-3 expression is especially found on T cells with suppressive functions, like Tregs^60^ and Tr1 cells,^61^ but can also be induced in innate immune cells.^58^ sLAG-3 can be generated by alternative splicing or cleavage from the membrane and was proposed to prevent intracellular LAG-3-mediated inhibitory signaling by interfering with ligand binding.^59^,^62^ However, there are also reports showing that cleavage of LAG-3 from the membrane is necessary for T cell proliferation and cytokine secretion, rendering sLAG-3 an inert by-product with short half-life.^63^ Interestingly, sLAG-3 can induce dendritic cells to secrete T_H_1 cytokines and stimulate antigen-specific CD8^+^ or T_H_1 responses when administered as adjuvant in mouse models.^64^–^66^ Increased LAG-3 expression of hepatic CD8 T cells was reported.^67^ Given that one of the best described LAG-3 ligands are peptide-loaded major histocompatibility complex II complexes,^58^,^59^ one may speculate that activated autoreactive T cells shed LAG-3, thus triggering a self-fueling inflammatory feedback loop by further activating antigen-presenting cells that desensitize the local hepatic environment to inhibitory signals and fuels AIH. This mechanism could also provide a functional link for the high association of AIH with distinct alleles of the *HLA-DRB1*-encoded beta subunit of major histocompatibility complex class II complexes.

This study has some limitations. The inclusion of patients with inadequate response and complete biochemical remission may appear as confounding factors for the discrimination of AIH from PBC. However, this patient heterogeneity reflects clinical reality in an outpatient setting and is actually no disadvantage in our approach which aimed to identify complementary markers for better categorization of patients with ambiguous serology at first presentation. The fact that some of the major discriminators identified here are not entirely independent of disease activity renders them especially valuable for disease monitoring. Furthermore, the applied definition of variant syndromes as PBC with moderate to severe periportal inflammation could result in misclassification of these patients as PBC because liver biopsy is infrequently performed in patients with PBC unless additional liver disease is suspected. In our cohort, 8 of 31 patients were biopsied and had only mild periportal inflammation (modified Hepatitis Activity Index≤4). As the majority of included patients with PBC had normal liver enzymes, it was not considered ethical to perform a liver biopsy on these. However, the mostly normal transaminase levels make it very unlikely that patients with significant periportal inflammation were included in the study.

Taken together, this study underpins T cell architectural changes and disease-specific immunoprofiles in variant syndromes of PBC and AIH that share features with the classical forms of these autoimmune liver diseases. The differential patterns of soluble factors and cytokines between AIH and PBC may shed light on some of the regulatory differences between these entities; however, their cellular sources and precise pathogenetic roles are yet to be determined. The intermediate profile observed in variant syndromes suggests that these may represent an immunological spectrum from AIH-like to PBC-like liver diseases reflected by their pattern of soluble immune checkpoint molecules and cytokines. Moreover, the immunogenic phenotypes we identified in this study were also associated with different clinical courses as patients with AIH-like variant syndrome were in a greater need of immunosuppressive therapy and but nevertheless tend toward disease progression. Therefore, the identification of the immunological fingerprint may identify risk patients in this hard-to-treat patient cohort and may also provide a basis for more individualized treatment in these sometimes difficult-to-manage patient groups.

## Supplementary Material

SUPPLEMENTARY MATERIAL
