# Supplementary figures and images for: Immune signatures in variant syndromes of primary biliary cholangitis and autoimmune hepatitis
Source: Hepatol Commun. 2023 Apr 26;7(5):e0123. doi: 10.1097/HC9.0000000000000123 (PMC10146553; doi:10.1097/HC9.0000000000000123)

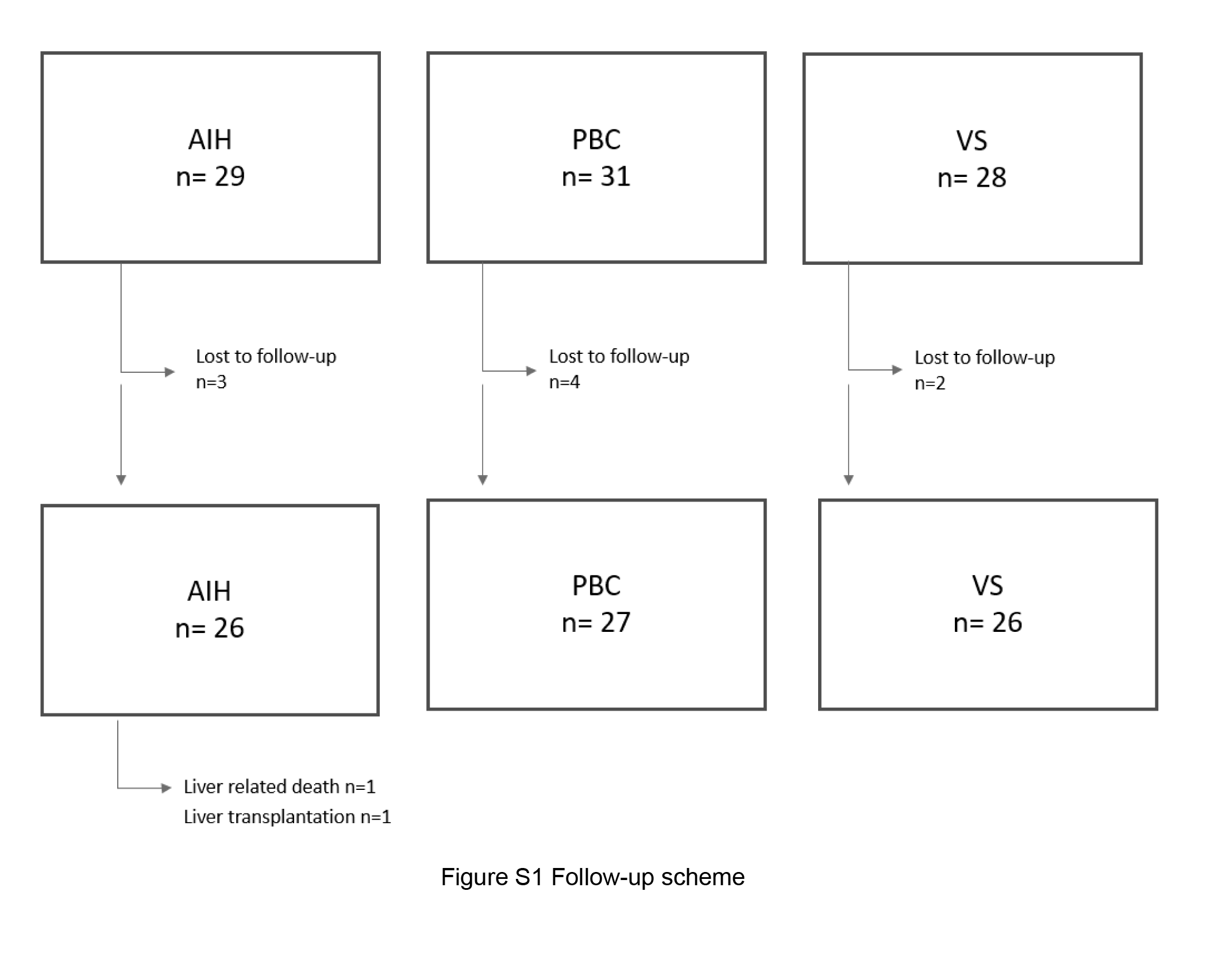

Supplement: SUPPLEMENTARY MATERIAL [file hc9-7-e0123-s001.tif]
